# Effect of Lamb and Soy Protein on Postprandial Glycaemic, Insulin and Appetite Responses to Potato, Pasta and Rice in Macronutrient-Equalised Meals—A Randomised Pilot Human Intervention Study

**DOI:** 10.3390/nu18142351

**Published:** 2026-07-17

**Authors:** Suman Sunita Mishra, Halina Stoklosinski, Duncan Hedderley, Hannah Dinnan, Sheridan Martell, Marian McKenzie, John Monro

**Affiliations:** Bioeconomy Science Institute, Private Bag 11600, Palmerston North 4442, New Zealand; halina.stoklosinski@plantandfood.co.nz (H.S.); duncan.hedderley@plantandfood.co.nz (D.H.); hannah.dinnan@plantandfood.co.nz (H.D.); sheridan.martell@plantandfood.co.nz (S.M.); marian.mckenzie@plantandfood.co.nz (M.M.); john.monro@plantandfood.co.nz (J.M.)

**Keywords:** lamb, soy, protein, glycaemic response, appetite

## Abstract

**Background:** Plant and animal proteins have been shown to affect postprandial glycaemic, insulin, and appetite responses to carbohydrate meals. Here we compared lamb protein and soy protein effects on glycaemic response, insulin secretion and appetite, examining whether the protein source modified the relative glycaemic effects of different carbohydrate foods, and whether lamb and soy protein differed in their effects. **Methods:** Healthy participants (*n* = 15) were randomised to consume meals containing potato, pasta, or rice, either alone or combined with equal amounts of lamb or soy protein, and with lamb fat content equalised across meals. Blood glucose and insulin responses were measured over the postprandial period, and subjective appetite was assessed. **Results:** Both lamb and soy protein reduced postprandial glycaemic responses and increased insulin responses compared with the carbohydrate staples consumed alone. Soy protein induced a slightly lower glycaemic response than lamb protein (132 ± 18 vs. 144 ± 13; 103 ± 16 vs. 123 ± 19; 112 ± 12 vs. 130 ± 17 mmol/L.min, potato, pasta and rice, respectively), although differences were small and not consistently significant. Adding protein also reduced the differences in glycaemic responses between the carbohydrate staples, particularly diminishing the higher response to potato. Both protein sources suppressed appetite to a similar extent relative to the carbohydrate-only meals. **Conclusions:** Adding protein to mixed meals had a dominant effect on postprandial glycaemic, insulin, and appetite responses, whereas the specific protein source (lamb versus soy) had a minor influence. These findings indicate that, under the conditions of this study, both animal and plant proteins were effective in moderating postprandial glycaemia and enhancing satiety.

## 1. Introduction

A number of studies have compared plant and animal proteins in terms of their effects on postprandial glycaemic and insulin responses to carbohydrate foods, with mixed findings. For instance, some have shown plant proteins to have a lower insulinotropic effect than whey protein [1] without increasing postprandial glycaemia. In contrast, wheat gluten was shown to be associated with a higher postprandial glycaemic response than whey, cod or casein in obese or diabetic subjects [1]. Another study comparing pea and rice proteins with whey protein found no difference between the plant proteins and whey in their effect on either the postprandial glycaemic or insulin responses [2]. Importantly, observed variations in post-meal blood sugar and insulin responses to plant- and animal-based proteins probably stem from diverse factors among protein sources. These include differences in amino acid makeup [3,4], digestion and absorption rates [4,5], and sample composition and processing levels [5,6], rather than simply the protein category.

There are several factors that complicate finding a general relationship between the effect of plant versus animal proteins on postprandial glycaemia and insulinaemia in mixed meals. The effect of the protein depends on the dose of protein ingested. It also depends on the amino acid composition of the protein, with proteins having a high leucine, isoleucine, valine, lysine and threonine content suppressing the glycaemic response. Co-consumed fat in a meal will also contribute to the suppression of glycaemic response, and its effect will depend on the composition of the fat. The degree of effect may also depend on the dose of available carbohydrate ingested with the protein.

If the protein effects on glycaemic response depend on the dose of a single type of carbohydrate, they should also depend on the digestibility of different available carbohydrates ingested at a single dose, because different rates of carbohydrate availability during digestion equate to different doses per unit time. This view is consistent with another study in which a salmon meal ingested with a staple carbohydrate (potato, rice or pasta) raised insulin response to above and reduced post-prandial glycaemic response to below that induced by the same amount of staple eaten alone [7]. However, the relative differences between the staples were reduced in the presence of the salmon. This suggests that staples, such as potatoes and pasta, which have very different glycaemic indexes (GIs), may not differ as much in glycaemic impact in a mixed meal context as responses to them alone, as in GI determination, suggest [8].

The above findings suggest that a protein with an insulinotropic effect will have a greater impact on a large glycaemic response than on a low glycaemic response. Furthermore, if different protein sources differ in their insulinotropic potency, the relative differences between the staples consumed with and without protein may depend on the type of protein consumed with the staple.

In a typical Western diet, potatoes are often consumed in a mixed meal with lamb. Potatoes have been cited as being generally of high GI [9] while pasta has been favoured for its low GI, and substitution of potatoes by pasta has been recommended. However, it is not known whether the glycaemic difference between potato and pasta on their own would be maintained in a mixed meal containing lamb protein. Furthermore, as far as we can ascertain, the impact of lamb protein on glycaemic and insulin responses to a carbohydrate load has not been experimentally determined.

Although potatoes have been viewed as of having a high GI they have the advantage of being of low energy density, and some research indicates that they are relatively satiating [10], perhaps because of their low energy density. Appetite is also suppressed by insulin, acting at the hypothalamus, so if lamb is insulinotropic it may combine well with potato to suppress appetite. And if lamb is more insulinotropic than soy protein, a typical plant protein, lamb may be more effective in suppressing glycaemic response and prolonging satiety than soy protein, particularly if paired with potato.

This paper reports results of research that has investigated the effects of lamb and soy proteins on glycaemic, insulin and satiety responses to potatoes, pasta and rice. We hypothesised that the proteins would suppress glycaemic response and enhance insulin response to the carbohydrate staples, that the effect would be greater for lamb than soy protein, and that the lamb would have a greater satiating effect than soy, particularly when ingested with potato.

## 2. Materials and Methods

### 2.1. Diet Preparation

#### 2.1.1. Ingredients

Potatoes of the variety Crop 17 marketed as Summer Delight were provided by Morgan Laurenson potato growers, Manawatu, New Zealand. The rice was white Kings Choice Basmati long-grain rice, and the pasta was Diamond macaroni made in Italy from durum wheat semolina, both purchased from Countdown Supermarket, Palmerston North, New Zealand. Lamb mince was obtained from The Mad Butcher, Palmerston North, New Zealand and consisted of minced hindquarter of New Zealand lamb. Lamb fat was rendered from offcuts of adipose tissue from lamb, also provided by The Mad Butcher. The sauce was Wattie’s garlic-flavoured pasta sauce, made in New Zealand and consisting of 87% tomatoes and 95% vegetables.

#### 2.1.2. Food Analyses

The moisture content of the meat was determined by freeze-drying triplicate subsamples in a vacuum oven at 80 °C for 20 h. The fat content of the mince was calculated as the loss in weight upon extracting fat from the freeze-dried mince by homogenising in a sequence of organic solvents including hexane, chloroform and finally acetone, with vacuum filtration in a sintered glass crucible at each stage and final drying of the acetone-washed residue at 100 °C. The weight of the dried residue was assumed to be protein.

The available carbohydrate content of the carbohydrate staples, potato, pasta and rice, was determined by in vitro digestion. The potatoes were scrubbed and boiled for 30 min by which time they were tender. The rice and pasta were cooked by boiling for 7 and 15 min, respectively, according to the packet instructions. All samples were subjected to a static in vitro digestion with pancreatin and amyloglucosidase, with samples removed for determination of available carbohydrate release at 0, 20, 40, 60, and 120 min. The samples were homogenised after the 120 min sampling and digested for a further 60 min with a further sample removed at 180 min. All samples (1 mL) were removed to 4 mL ethanol, mixed, centrifuged, and an aliquot of the supernatant was subjected to a secondary amyloglucosidase digestion before determining reducing sugars. Available carbohydrate was therefore defined as the 80% ethanol-soluble carbohydrates released up to 180 min of amylolysis of the staples.

#### 2.1.3. Preparation of Meals

The amount of lamb required to provide 30 g of protein and the amount of each staple required to provide a 40 g dose of available carbohydrate were calculated and multiplied by the number of meals required for the trial. The required amount of each staple was cooked as one batch and then divided into the number of meals required, taking into account the change in weight upon cooking. The lamb mince was gently fried until cooked through without browning and combined with enough pasta sauce to make the meal palatable. To each of the meals not containing lamb, 12.6 g of rendered lamb fat was added so that all meals contained the same quantity of pasta sauce and lamb fat (Table 1).

### 2.2. Human Intervention Study

The trial was run as a non-blinded, randomised, repeated measures study as it was not possible to blind the subjects to the meals they were consuming. Randomisation was achieved using a Williams Latin Square design.

#### 2.2.1. Recruitment and Screening

**Figure 1:**
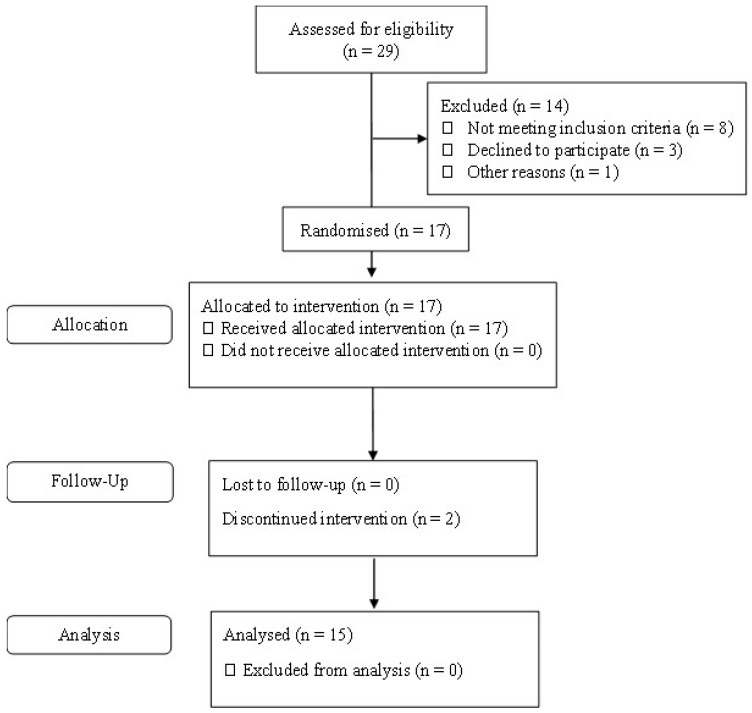
CONSORT diagram for study.

Fifteen healthy volunteers (18–65 y) were recruited using a flyer and email that briefly described the study, with pre-screening by phone. The volunteers were asked initial recruitment questions to determine their suitability to take part in the study (Figure 1). The nature of the study and their involvement and responsibilities were described to them. Volunteers deemed suitable were sent an information sheet containing study details, and an informed consent form. Volunteers who were willing to participate were then called to the clinic. A blood sample was taken for measurement of fasting blood glucose and glycosylated haemoglobin (HbA1c). Anthropometric measurements were also taken (Table 2). This first visit was also used to familiarise participants with the blood sampling procedure they would be subjected to as volunteers in the study. The health of the volunteers was gauged by self-assessment and their score on the General Health Questionnaire. Exclusion criteria were as follows: glucose intolerance (any history of diabetes or evidence of glucose intolerance in the preliminary test of fasting blood glucose and HbA1c), pregnancy or breastfeeding, being vegan or vegetarian, being allergic to or intolerant of potatoes, pasta, rice, plant protein or meat, taking any antacids, laxatives or supplements, and recent ill health. The participants were recruited from Palmerston North and the trial was carried out at the Bioeconomy Science Institute’s clinical facility in Palmerston North.

#### 2.2.2. Study

In preparation for each testing session, participants were asked to fast from 10.00 p.m. the night before a test with water consumption not restricted, consume a similar evening meal before each test session, avoid strenuous physical activity and refrain from smoking or consuming alcohol the evening before a test and on the day of the test.

On each test day, the volunteers were seated and asked to remain so for the duration of the test. On arrival, the participants were asked to relax for 15 min before baseline blood sugar measurements were taken in duplicate. The participant was then given a test meal by the research staff and instructed to consume the whole amount within a 10 min period. Blood glucose testing was timed from the start of food consumption by finger prick sampling of capillary blood at 15 min intervals in the first hour and then at 30 min intervals until 150 min had elapsed. Samples were thus collected at 0 (baseline × 2), 15, 30, 45, 60, 90, 120, and 150 min and blood glucose was measured immediately using a HemoCue^®^ blood glucose meter (HemoCue AB, Ängelholm, Sweden). For insulin analysis, a capillary blood sample was drawn into a Lithium Heparin (LH) Microvette^®^ microtube (CB 300 LH, Sarstedt AG & Co., Nümbrecht, Germany), capped, inverted and stored on ice for a maximum of 1 h before centrifugation. Tubes were centrifuged at 2000× *g* for 15 min at 4 °C. Using a filter tip, 50 µL of plasma was aliquoted into a 1.5 mL Eppendorf tube, placed in a −20 °C freezer and subsequently stored at −80 °C until analysis.

#### 2.2.3. Human Insulin

Plasma insulin was quantified following the assay procedure of the Human Insulin ELISA kit supplied by EMD Millipore Corporation, Burlington, MA, USA (Cat. # EZHI-14K). Absorbance was read at 450 nm on a FLUOStar Optima^®^ Platereader (BMG Labtech, Mornington, VIC, Australia). A 4-parameter logistic (4PL) standard curve was fitted for each ELISA plate and used to calculate insulin concentrations of the unknown plasma samples.

#### 2.2.4. Satiety

The subjects were asked to rate their appetite at 0, 60 and 150 min using a four-dimensional, 10 cm, visual analogue scale (VAS), with the dimensions: How hungry do you feel? (Not at all hungry—extremely hungry); How full do you feel? (Not at all full—extremely full); How strong is your desire to eat? (Not at all strong—extremely strong); How much food do you think you can eat? (Nothing at all—a large amount) [11]. The area under the curve (cm.min) for each dimension was calculated.

A composite appetite score was also calculated as the average of Hunger, 10-Fullness, Desire to eat, and Amount that could be eaten.

The research in this project was undertaken in a culturally sensitive manner, with all aspects of the trial explained clearly and appropriately to the participants. Researchers were available to answer questions throughout the study and seek advice from appropriate advisory groups should it be necessary. The opportunity for whānau (family) support was available at all times. Participants were allowed to consult with other people, such as whānau or healthcare providers. Participants were allowed to bring family, whānau, and friends for support during the trial (as is suggested in the New Zealand ethics application). Participants were allowed to withdraw from the trial at any point with no questions asked. Participants were given a “koha” token of appreciation for taking part in the trial.

The human intervention study was approved by the Human and Disabilities Ethics Committee of the New Zealand Ministry of Health, and the trial was registered with the Australia New Zealand Clinical Trials Registry (Trial ID: ACTRN12619000777190, 24 May 2019).

Informed consent was obtained from all subjects involved in the study. The trial was conducted between 15th July 2019 and 21 July 2020.

#### 2.2.5. Data Analysis

Incremental blood glucose responses were calculated by subtracting each individual’s baseline value from subsequent measurements. The incremental values were then used to determine the positive incremental area under the curve (IAUC) for each individual. GenStat software was used (version 11.1, VSNi Ltd., Hemel Hempstead, UK) in an unbalanced analysis of variance (ANOVA), testing differences between meals after adjusting for participant and order effects. Power calculation—normally, 10 participants are recruited for GI testing. In this trial, 15 participants were recruited.

## 3. Results

All participants recruited for the study were in good health, and showed no sign of metabolic disorders involving glucose intolerance (Table 2).

The estimated proximate composition of the meals showed that the protein meals, the major focus of this paper, were similar in protein (range 33.7–37.1 g), carbohydrate (range 44.1–44.6 g), fat (12.2–13.9 g) and energy (1714–1961 kJ).

**Figure 2:**
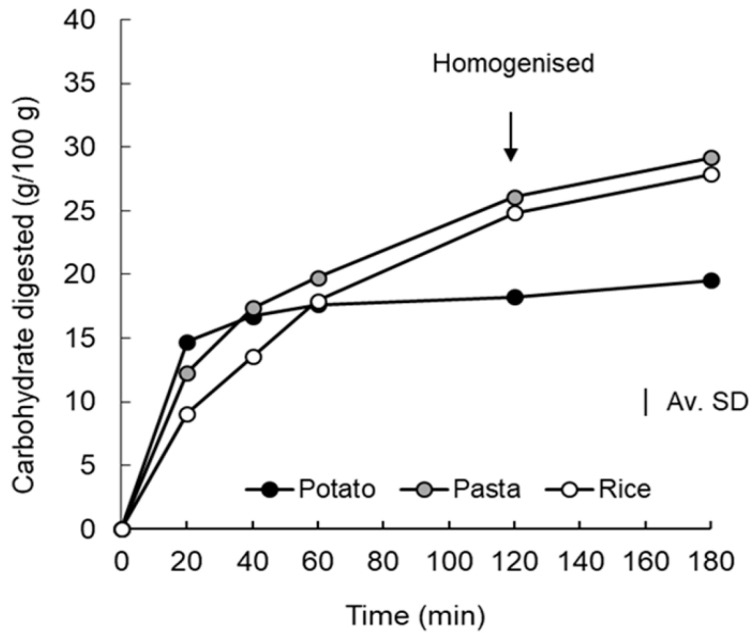
In vitro digestive analysis of carbohydrate staples showing the contrasting profile of potato compared with pasta and rice. Means of triplicate determinations.

The in vitro analysis of available carbohydrates in the carbohydrate staples, potato, pasta and rice provided available carbohydrate data for the meal formulation and revealed differences in the digestibility of carbohydrates in the staples. Rapidly digested carbohydrate was greatest for the potato (14.7 g/100 g) and less for the pasta (12.3 g/100 g) and rice (9.1 g/100 g). However the potato digestion reached a plateau before the pasta and rice, so that at 120 min substantially more carbohydrate had been digested from the pasta (26.1 g/100 g) and rice (24.8 g/100 g) (Figure 2).

The release of carbohydrate at 20 min (“rapidly available carbohydrate”) is an indicator of relative glycaemic impact and, expressed as a percentage of total available carbohydrate (release at 180 min), it provides an estimate of GI: potato 75%, pasta 42%, and rice 33% in the present case. Corresponding published values are potato (Nadine, New Zealand) 70 ± 17, macaroni 47 ± 2 (mean of 2 studies), and rice (long grain) 56 ± 2 [12].

**Figure 3:**
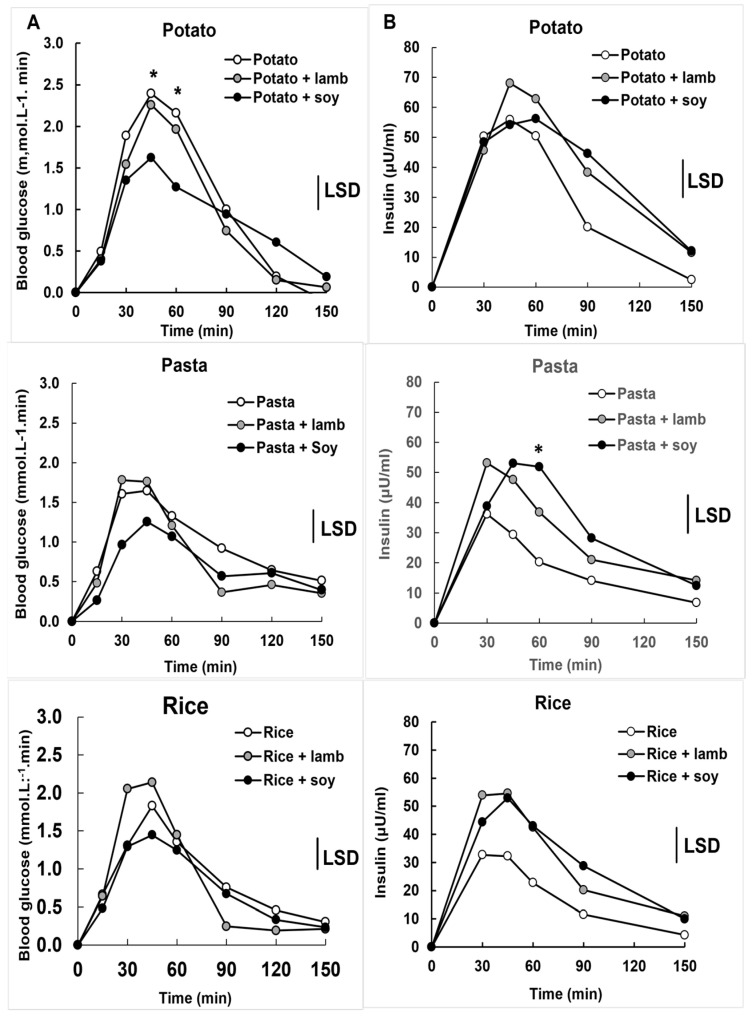
Blood glucose (**A**) and insulin (**B**) responses to potato, pasta, and rice alone and in the presence of soy protein or lamb. (Mean and least significant differences shown). Sources of significant difference given in Table 3. Asterix shows time of significant difference.

The blood glucose responses to the staples reflected their relative digestibilities in vitro. The relative values for rapidly digested carbohydrate in vitro were potato: pasta: rice, 1:0.84:0.62, while in vivo they were 1:0.89:0.80 for iAUC and 1:0.69:0.35 for peak height (Figure 3 and Table 3 and Table 4). The potato was clearly the most glycaemic and most insulinogenic of the three staples tested.

**Figure 4:**
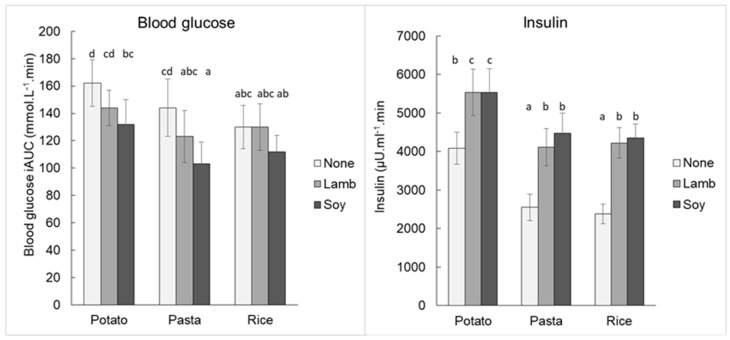
Sources of significant difference in incremental blood glucose (BGR) (iAUC) and insulin (AUC) responses to carbohydrate staples consumed with and without soy protein and lamb. Bars with a letter in common within each starch source are not significantly different.

Soy protein consumed with all three staples reduced blood glucose peak height compared with lamb, but also attenuated the glycaemic response, so that differences between soy protein and lamb in areas under the curve were not significant (Table 3). However, soy protein with potato and pasta significantly suppressed glycaemic response (iAUC) compared with potato and pasta alone, whereas lamb did not (Figure 4). The mean percentage changes in glycaemic response (iAUC) as a result of consuming protein with the carbohydrate foods were potato (soy −18.5%, lamb −11.1%), pasta (soy −28.5%, lamb −14.6%) and rice (soy −18%, lamb 0%).

Linear mixed-effect models were fitted to the iBGR and iIR data, with fixed effects for diet, time and diet × time, a random effect for participant, and auto-correlated errors using a power model to account for some observations being closer than others.

For iBGR, there was a significant effect of time (F = 196.5 on 7 and 815 df, *p* < 0.001), and a significant diet × time interaction (F = 3.3 on 56 and 882 df, *p* < 0.001) but no significant diet main effect (F = 1.3 on 8 and 180 df, *p* = 0.242). Using Tukey’s test to compare the three types of protein within each starch at each time point, there were significant differences for the diets with potato at 45 and 60 min, with potato + soy significantly lower than potato alone.

For iIR, there was a significant effect of time (F = 192.9 on 5 and 611 df, *p* < 0.001), a significant effect of diet (F = 10.4 on 8 and 311 df, *p* < 0.001), and a significant diet × time interaction (F = 2.7 on 40 and 676 df, *p* < 0.001). Using Tukey’s test to compare the three types of protein within each starch at each time point, there was a significant difference for the diets with pasta at 60 min, with pasta + soy significantly higher than pasta alone.

Figure 4 illustrates the Diet effect for iIR. Average insulin level tended to be higher with a protein than with a starch alone, and the difference was more marked for rice and pasta.

The incremental blood glucose peak heights at 60 min, at which time measurement of appetite was made, were potato 2.16, pasta 1.21 and rice 1.45 mmol/L. The response to potato was significantly (LSD 0.43) higher than for pasta and rice, by 79% and 49%, respectively (Table 4).

In contrast, both lamb and soy protein with all carbohydrate sources significantly and similarly increased the insulin response compared with the carbohydrates consumed alone (Figure 4). The mean percentage increases in insulin response as a result of consuming protein were greater for pasta (soy 75.8%, lamb 61.3%) and rice (soy 83.1%, lamb 77.7%) than for potato (soy 35.6%, lamb 35.5%), which was significantly more insulinogenic on its own than the more slowly digested pasta and rice (Figure 3).

There was no significant interaction between carbohydrate and protein in effects on blood glucose or insulin response (Table 3).

**Figure 5:**
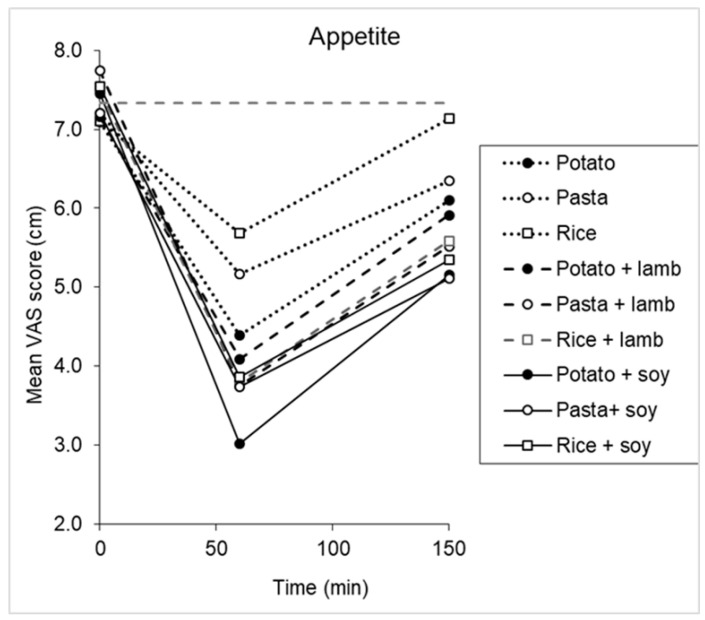
Effect of lamb and soy protein consumed with carbohydrate staples (potato, pasta, rice) on appetite expressed as the mean score on four dimensions of appetite—hunger, 10-fullness, desire to eat and quantity that could be eaten.

All the meals consumed had a definite effect of suppressing appetite on all dimensions, with the greatest suppression at 60 min and partial recovery by 150 min (Figure 5). A composite appetite variable calculated as the mean of all four dimensions (with Fullness converted to a 10-Fullness score) correlated closely with all dimensions of appetite, which were themselves closely correlated, and was used to clearly represent the change in appetite in response to all nine meals over time (Table 5 and Table 6).

**Figure 6:**
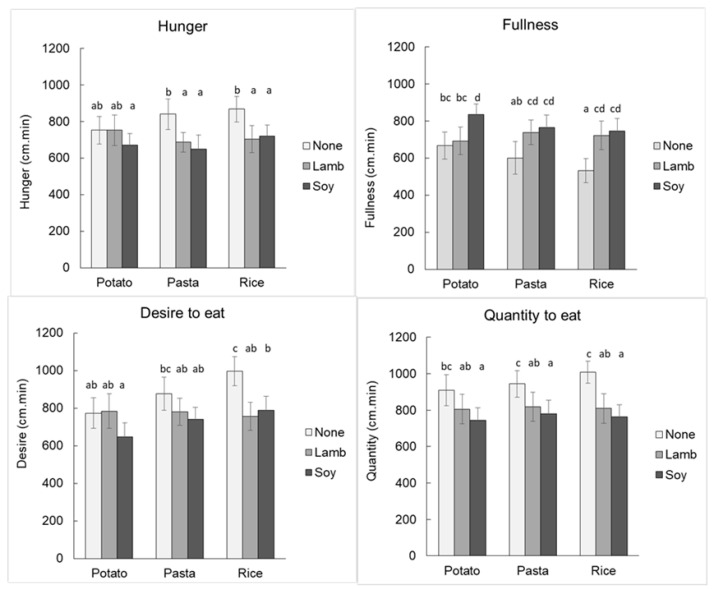
Effect of lamb and soy protein on appetite responses to carbohydrate staples potato, pasta and rice. Bars with a letter in common within each starch source are not significantly different (*p* < 0.05).

Differences between the carbohydrate staples consumed alone in their effect on appetite were small and generally non-significant, although potatoes tended to suppress appetite most, and rice least. However, both protein sources significantly and similarly suppressed appetite compared with the staples consumed without protein. Lamb and soy were, however, very similar in their effects on appetite (Figure 6).

## 4. Discussion

The research questions addressed in the present study were:

- Does lamb protein differ from soy protein in its effects on glycaemic response?
- Are lamb and soy proteins equally insulinogenic?
- Are the relative glycaemic effects of different carbohydrate sources affected by animal versus plant protein?
- Does lamb protein differ from soy protein in its effects on satiety?

The presence of protein influenced the relative glycaemic response of carbohydrate staples. On their own, potatoes elicited a significantly higher glycaemic response (162 ± 17 mmol/L.min) compared to pasta (144 ± 21 mmol/L.min) and rice (130 ± 16 mmol/L.min) because they digest more quickly. Nonetheless, these differences decreased when meals included lamb or soy protein. Soy protein tended to suppress glycaemic response more strongly than lamb protein, but not always significantly more than lamb, which also suppressed glycaemic response to potato and pasta. It is interesting to note that the marginally greater glycaemic suppression by soy protein than lamb was not reflected in a much greater insulin response to soy than to lamb. Similar results have been observed by others—a randomised, crossover study comparing pea protein with whey protein found that pea protein slightly lowered blood glucose more than whey, with insulin responses following a similar pattern [13]. These findings suggest that the minor difference in blood sugar reduction with soy protein may not be solely due to differences in insulin secretion. It might also involve factors like digestion rate, gastric emptying, or peripheral glucose absorption. However, in the present study, equalisation of lamb fat across all diets would have removed any influence of animal fat on gastric emptying.

This study aligns with other research on mixed meals. For example, consuming salmon with potatoes, rice, or pasta has been shown to increase insulin responses and reduce postprandial blood sugar levels compared to eating the same carbs alone. These studies also indicate that the GI of foods, when measured in isolation, does not consistently predict their impact within a mixed meal. Overall, the findings suggest that post-meal blood sugar levels are mainly influenced by the entire meal composition rather than the characteristics of individual carbohydrate foods.

Although the lamb or soy protein added to the staple meals did not differ greatly in their effects on glycaemic and insulin responses, both protein sources substantially and similarly increased plasma insulin concentrations compared with the staples consumed without protein. This is consistent with the firmly established insulinotropic effect of dietry proteins [14]. Where differences in the glycaemic and insulinogenic effects of animal and soy proteins have been found, they have been attributed to the difference in amino acid composition of animal and plant proteins. To our knowledge, this is the first study of this type carried out on New Zealand lamb, which is grass-fed, so they may vary in amino acid composition. Protein ingestion stimulates insulin secretion both directly, through circulating amino acids, and indirectly via incretin hormones [3]. Meta-analysis evidence has demonstrated that incorporating protein into carbohydrate meals enhances insulin responses while reducing postprandial glycaemia [5]. In the current study, insulin responses to lamb and soy were quite similar, and the macronutrient quantities in the protein diets were matched, suggesting that differences in amino acid composition between lamb and soy proteins did not significantly affect insulinogenic potency. The macronutrient (protein, carbohydrate, fat) contents of the protein diets were approximately the same (Table 1) so any differences in response would be due to intrinsic differences between the proteins (protein “quality”) or to other food factors, rather than protein quantity.

The lamb and soy proteins substantially suppressed appetite compared with the staples consumed without protein [15,16], but their effects were very similar in degree [17]. The meals were balanced with respect to fat, carbohydrate, and total mass (with water), and the protein meals contained approximately the same amount of protein. The suppression of appetite by protein may have been due to the mass of protein retained by the stomach [18], and perhaps due to the influence of insulin, which has an appetite-suppressing effect. As blood glucose was not higher in the presence of protein it is unlikely to have played a role in the appetite-lowering effects of the proteins [17].

However, blood glucose concentration may have influenced the difference in appetite responses to the staple meals consumed alone (i.e., without added protein), because at 60 min the blood glucose response to potato was substantially higher than for pasta and rice by 79% and 49%, respectively. These values reflect the high in vitro digestion rate of potato compared with pasta and rice. Potato has been shown to have a more satiating effect than pasta and rice [19], and it has been suggested that a lower energy (available carbohydrate) density could have been responsible. But in the present experiment the participants received a supplement of water that would have equalised the carbohydrate density in the staple meals without added protein.

This study has certain limitations. As a pilot study the amount of data generated by it did not allow a wide range of conclusions or secondary outcomes to be addressed. It examined only immediate post-meal responses, so the results might not reflect long-term effects. While the amount of protein and fat was controlled, the study did not analyse variations in amino acid profiles. The findings may not be applicable to individuals with impaired glucose regulation. Moreover, appetite was assessed subjectively without measuring subsequent energy intake or appetite hormones.

A number of physiological factors involved in glucose homeostasis were intentionally not included in this study, because it was a pilot study without the resources for an in-depth study of mechanisms. Such factors would be appropriate to include in further studies based on the pilot study results, depending on the hypotheses being tested. Future studies should investigate long-term effects, underlying mechanisms such as hormonal responses and gastric emptying, and responses in physiologically different populations.

## 5. Conclusions

In conclusion, adding protein to carbohydrate meals significantly affected post-meal blood glucose levels, insulin release, and appetite. The specific type of protein—whether lamb or soy—had minimal impact. Both protein sources similarly reduced blood glucose responses, increased insulin secretion, and reduced appetite. These findings indicate that overall protein intake, rather than protein type, primarily influences post-meal metabolic and appetite responses in this study.

## Figures and Tables

**Table 1:** Composition of meals.

**A. Food Components in the Nine Test Meals (g)**									
	**1**	**2**	**3**	**4**	**5**	**6**	**7**	**8**	**9**
	**Potato**	**Potato + Lamb**	**Potato + Soy**	**Pasta**	**Pasta + Lamb**	**Pasta + Soy**	**Rice**	**Rice + Lamb**	**Rice + Soy**
Potato *	205	205	205	-	-	-	-	-	-
Pasta *	-	-	-	137	137	137	-	-	-
Rice *	-	-	-	-	-	-	144	144	144
Lamb	-	158	-	-	158	-	-	158	-
Soy protein	-	-	33	-	-	33	-	-	33
Lamb fat	12.3	-	12.3	12.3	-	12.3	12.3	-	12.3
Pasta sauce	44	44	44	44	44	44	44	44	44
Water **	146	0	113	214	68	181	207	61	174
**B. Major Macronutrients per Meal in the Nine Test Meals**									
	**1**	**2**	**3**	**4**	**5**	**6**	**7**	**8**	**9**
	**Potato**	**Potato + Lamb**	**Potato + Soy**	**Pasta**	**Pasta + Lamb**	**Pasta + Soy**	**Rice**	**Rice + Lamb**	**Rice + Soy**
Energy (kJ)	1247	1714	1801	1406	1874	1961	1265	1733	1820
Protein (g)	5.0	33.8	35.1	7.0	35.8	37.1	4.9	33.7	35.0
Fat (g)	12.8	12.2	13.8	13.1	12.5	14.1	12.9	12.3	13.9
Carbohydrate (g)	44.1	44.1	44.4	44.1	44.1	44.4	44.2	44.2	44.6

* Cooked. ** Consumed separately with meal.

**Table 2:** Participant characteristics.

Anthropometric Data for the Participants	
Sex (male:female) *n*:*n*	8:7
Ethnicity (Caucasian:Asian:Persian) *n*:*n*:*n*	12:2:1
Age, y	42.4 ± 3.7
Height, m	1.72 ± 0.21
Weight, kg	75.2 ± 5.2
Body mass index (BMI), kg/m^2^	25.0 ± 1.2
Fasting plasma glucose, mmol/L	4.4 ± 0.1
Glycosylated haemoglobin (HbA1c), mmol/mol	32.1 ± 0.9

**Table 3:** Sources of significant difference in blood glucose (BGR) and insulin responses to carbohydrate staples consumed with and without soy protein and lamb. (Results are plotted in Figure 3).

			BGR iAUC (mmol/L.min)			Insulin (μU/mL.min)		
Protein	Carbohydrate	n	Mean	s.e.		Mean	s.e.	
None	Potato	15	162	17	d	4082	419	b
	Pasta	15	144	21	cd	2548	337	a
	Rice	15	130	16	abc	2378	251	a
Lamb	Potato	15	144	13	cd	5532	604	c
	Pasta	15	123	19	abc	4110	485	b
	Rice	15	130	17	abc	4225	398	b
Soy	Potato	15	132	18	bc	5535	620	c
	Pasta	15	103	16	a	4479	516	b
	Rice	15	112	12	ab	4353	368	b
LSD			28			843		
ANOVA			F	*p*		F	*p*	
Carbohydrate	2 and 112 df		4.9	0.009		20.7	<0.001	
Protein	2 and 112 df		6.6	0.002		32.3	<0.001	
Carbohydrate × protein	4 and 112 df		0.4	0.784		0.3	0.88	

Means with a letter in common within a column are not significantly different (*p* < 0.05).

**Table 4:** Sources of significant difference in amplitude of incremental blood glucose (BGR) and insulin responses to carbohydrate staples consumed with and without soy protein and lamb, for each person on each diet.

			BGR (mmol/L)			Insulin (μU/mL)		
Protein	Carbohydrate	n	Mean	s.e.		Mean	s.e.	
None	Potato	15	2.8	0.20	e	66.8	6.3	bc
	Pasta	15	1.95	0.16	bc	41.6	7.0	a
	Rice	15	1.87	0.19	bc	38.8	4.8	a
Lamb	Potato	15	2.38	0.15	d	74.2	7.4	c
	Pasta	15	2.09	0.18	cd	61.8	8.0	bc
	Rice	15	2.31	0.18	d	65.2	65.8	bc
Soy	Potato	15	1.87	0.16	bc	67.1	6.1	bc
	Pasta	15	1.40	0.16	a	62.0	8.4	bc
	Rice	15	1.65	0.12	ab	59.8	5.1	b
LSD			0.34			12.5		
ANOVA			F	*p*		F	*p*	
Carbohydrate	2 and 112 df		11.9	<0.001		7.0	<0.001	

Means with a letter in common within a column are not significantly different (*p* < 0.05).

**Table 5:** Correlations between mean scores for appetite dimensions across nine test meals and a composite appetite dimension (All mean) calculated as the mean of the four dimensions scored.

	Hunger	10-Fullness	Desire	Quantity	All Mean
Hunger	-	0.98	0.89	0.90	0.96
10-Fullness			0.95	0.95	0.99
Desire				0.87	0.95
Quantity					0.95

**Table 6:** Statistical significance (ANOVA) of effects of carbohydrate and protein on dimensions of appetite measured by visual analogue scale.

		Hunger		Fullness		Desire		Quantity	
ANOVA	df	F	*p*	F	*p*	F	*p*	F	*p*
Carb.	2 and 112	0.83	0.441	1.7	0.186	3.9	0.023	0.92	0.403
Protein	2 and 112	8.9	<0.001	13.8	<0.001	7.9	<0.001	21.6	<0.001
Carb. × protein	4 and 112	1.3	0.274	1.1	0.349	1.7	0.148	0.6	0.661

## Data Availability

The original contributions presented in this study are included in the article. Further inquiries can be directed to the corresponding author.
